# Steroid Receptor Coactivator-3 (SRC-3/AIB1) as a Novel Therapeutic Target in Triple Negative Breast Cancer and Its Inhibition with a Phospho-Bufalin Prodrug

**DOI:** 10.1371/journal.pone.0140011

**Published:** 2015-10-02

**Authors:** Xianzhou Song, Chengwei Zhang, Mingkun Zhao, Hui Chen, Xing Liu, Jianwei Chen, David M. Lonard, Li Qin, Jianming Xu, Xiaosong Wang, Feng Li, Bert W. O’Malley, Jin Wang

**Affiliations:** 1 Department of Pharmacology, Baylor College of Medicine, Houston, Texas, United States of America; 2 Department of Molecular and Cellular Biology, Baylor College of Medicine, Houston, Texas, United States of America; 3 Lester & Sue Smith Breast Center, Baylor College of Medicine, Houston, Texas, United States of America; 4 Center for Drug Discovery, Baylor College of Medicine, Houston, Texas, United States of America; 5 Integrative Molecular and Biomedical Sciences Graduate Program, Baylor College of Medicine, Houston, Texas, United States of America; Florida International University, UNITED STATES

## Abstract

Triple negative breast cancer (TNBC) has the poorest prognosis of all types of breast cancer and currently lacks efficient targeted therapy. Chemotherapy is the traditional standard-of-care for TNBC, but is frequently accompanied by severe side effects. Despite the fact that high expression of steroid receptor coactivator 3 (SRC–3) is correlated with poor survival in estrogen receptor positive breast cancer patients, its role in TNBC has not been extensively investigated. Here, we show that high expression of SRC–3 correlates with both poor overall survival and post progression survival in TNBC patients, suggesting that SRC–3 can serve as a prognostic marker for TNBC. Furthermore, we demonstrated that bufalin, a SRC–3 small molecule inhibitor, when introduced even at nM concentrations, can significantly reduce TNBC cell viability and motility. However, because bufalin has minimal water solubility, its *in vivo* application is limited. Therefore, we developed a water soluble prodrug, 3-phospho-bufalin, to facilitate its *in vivo* administration. In addition, we demonstrated that 3-phospho-bufalin can effectively inhibit tumor growth in an orthotopic TNBC mouse model, suggesting its potential application as a targeted therapy for TNBC treatment.

## Introduction

Triple negative breast cancer (TNBC) accounts for ~15–20% of breast cancer incidence [1]. These tumors are highly aggressive, metastatic, and have the worst short-term prognosis among all breast cancer types [1,2]. Unlike estrogen receptor positive (ER+) breast cancer, which can be effectively treated with aromatase inhibitors, tamoxifen or other selective estrogen receptor modulators (SERMs), there is no targeted therapy currently approved for TNBC. Chemotherapy and surgery are the current standard-of-care (SOC) for TNBC [1,3,4]. Besides the severe side effects associated with chemotherapies, the selection pressure induced by nonspecific chemotherapy drugs and the development of drug resistance even can promote metastasis [5–8]. Surgical removal of the primary tumor also may promote proliferation of metastases, in part due to excessive release of growth factors intended for wound healing [9–11]. Targeted therapy is limited because the vast majority of inhibitors can inhibit only one pathway. Consequently, drug resistance occurs when tumors use alternative growth signaling pathways, subverting the original therapeutic target. Because of this, there is an urgent need to identify applicable targets and to develop novel treatments for either pre-surgical neoadjuvant therapy or in combination with SOC to improve TNBC treatment outcomes.

Steroid receptor coactivator 3 (SRC–3, also known as AIB1, NCOA3, ACTR, pCIP, RAC3, and TRAM1) is one of three homologous members of the p160 SRC family [12–14]. As a master regulator of cellular growth and organism development, SRC–3 sits at the nexus of many intracellular signaling pathways that are critical for cancer proliferation and metastasis. It is well established that SRC–3 plays a pro-proliferative role in primary tumor formation in the mammary gland [15]. SRC–3 knockout protected mice from oncogene and chemical carcinogen induced mammary gland tumorigenesis [16,17]. SRC–3 also regulates cell motility, invasion and tumor metastasis. Absence of SRC–3 in a transgenic breast cancer mouse model substantially reduced mammary tumor metastasis to the lung [18]. Therefore, our prior studies demonstrate that inhibition of SRC–3 can impact many cancer signaling pathways simultaneously. It is critical to note that down-regulation or inhibition of SRC–3 in normal cells at moderate doses does not influence adult mice life span [19]. Due to these favorable features discussed above, SRC–3 is an ideal target for prospective TNBC therapeutics.

Overexpression of SRC–3 was linked to clinical and experimental endocrine resistance of ER+ breast cancer [20–22]. However, the role of SRC–3 in TNBC has not yet been investigated. Prior to initiating our study, we analyzed the KM-plotter database [23] and noted that SRC–3 expression levels negatively correlated with the overall survival of TNBC patients. Therefore, we believe that SRC–3 could be a promising target for this subset of breast cancer cases.

Identifying small molecule inhibitors (SMIs) for SRC–3 is challenging because SRC–3 is a large and mostly non-structured nuclear protein (32). We initially reported gossypol as a “proof-of-concept” SRC–3 SMI [24]. Despite the encouraging success of gossypol as the first selective SRC–3 SMI, its IC_50_ values are micromolar, which is suboptimal for drug development and can create off-target toxicity [24]. Taking advantage of high throughput screening, we found a series of cardiac glycosides (CGs) that can function as SRC–3 SMIs [25]. The most potent candidate, bufalin, directly binds to SRC–3 in its receptor interacting domain and selectively reduces the levels of SRC–3 in ER+ breast cancer cell lines without perturbing overall protein expression patterns [25]. Additionally, bufalin exhibits IC_50_ values in the low nM range with selective toxicity towards cancer cells, keeping normal cell viability unperturbed [25–27].

However, the low solubility of bufalin has limited its development as a viable therapeutic agent. In our previous study, we utilized nanotechnology to facilitate bufalin delivery and achieved inhibition of tumor growth *in vivo*.[25] In this study, we explored an alternative prodrug strategy that converts water insoluble bufalin into a soluble analog: 3-phospho-bufalin (p-Buf). P-Buf can be hydrolyzed by endogenous phosphatases under physiological conditions to regenerate bufalin. This prodrug strategy avoids sudden exposure to high concentrations of free bufalin, which may cause acute cardiotoxicity. In addition, we demonstrate that p-Buf can significantly reduce tumor growth in an orthotopic TNBC model.

## Materials and Methods

### Bioinformatics Analyses of SRC–3 Levels in TNBC Patients

The bioinformatics analyses of SRC–3 levels in TNBC patients were performed on kmplot.com [23]. The Affymetrix probe 209061_at (NCOA3) was used for analyses. The groups with high and low SRC–3 expression levels were determined using “auto select best cutoff”. Patient cohort with the intrinsic basal subtype was included for all the analyses, including overall survival, distant metastasis free survival, and post progression survival. The 2014 version (n = 4142) of the database was chosen. The detailed parameters used for the bioinformatics analyses can be found in Table 1.

**Table 1:** Parameters used for bioinformatics analyses on kmplot.com.

Figures	1A	1B	1C	1D
**Affymetrix ID**	209061_at NCOA3	209061_at NCOA3	209061_at NCOA3	209061_at NCOA3
**Survival**	OS	OS	DMFS	PPS
**Auto select best cutoff**	Checked	Checked	Checked	Checked
**Follow up threshold**	All	All	All	All
Censore at threshold	Checked	Checked	Checked	Checked
**Compute median over entire database**	FALSE	FALSE	FALSE	FALSE
**Cutoff value used in analysis**	470	470	590	494
**Expression range of the probe**	155–1977	155–1978	135–1977	230–975
**Probe set option**	User selected	User selected	User selected	User selected
**Invert HR values below 1**	Not checked	Not checked	Not checked	Not checked
**Restrictions**				
ER status	All	All	All	All
derive ER status from gene expression data	Not checked	Not checked	Not checked	Not checked
PR status	All	All	All	All
HER2 status	All	All	All	All
Lymph node status	All	All	All	All
Intrinsic subtype	Basal	Basal	Basal	Basal
TP53 status	All	All	All	All
Grade	All	All	All	All
Use earlier release of the database	2014 version (n = 4142)	2014 version (n = 4142)	2014version (n = 4142)	2014version (n = 4142)
Use following dataset for the analysis	All	All	All	All
**Quality control**				
Remove redundant samples	Checked	Checked	Checked	Checked
Array quality control	Exclude biased arrays	Exclude biased arrays	Exclude biased arrays	Exclude biased arrays
Proportional hazards assumption	Checked	0.1267	Checked	0.2506
**Cohort**				
Cohorts	Not Selected	Untreated patients	Not Selected	Not Selected
**Results**				
P values	0.029	0.0245	0.1647	0.0045

### Cell Culture

Triple negative breast cancer (TNBC) cell lines HCC1143, SUM149PT, SUM159PT and MDA-MB–231 were obtained from ATCC. The MDA-MB-231-LM3-3 (LM3-3) cell line was developed from lung metastasis derived from a MDA-MB-231-LM2 xenograft tumor in the mammary fat pad of a SCID mouse [28].

The HCC1143 cell line was grown in RPMI–1640 medium, SUM149PT and SUM159PT cell lines were grown in F–12 medium, and MDA-MB–231 cells were grown in DMEM. All culture media were supplemented with 10% fetal bovine serum. All cell lines were grown in a humidified incubator containing 5% CO_2_ at 37°C. Primary hepatocytes were isolated from an adult male mouse (C57BL) by collagenase perfusion (0.8 mg/mL; Sigma–Aldrich, C5138) through the portal vein as described previously [29], and maintained in M199 medium with 10% FBS. The viability of freshly isolated hepatocytes was verified using Trypan blue staining.

### Cell Cytotoxicity Assays

TNBC cells (5000 to 7000 cells per well) were seeded in 96-well plates in medium supplemented with 10% FBS. On the next day, when cells reached 70% to 80% confluence, bufalin (Santa Cruz) was added to achieve an array of final concentrations (0.2, 0.5, 1, 2, 5, 10, 20, 50, 100, 500 and 1000 nM). After 72 hours of bufalin treatment, cell viability was measured by MTT assay. Cell viabilities relative to untreated control cells were plotted using Graphpad Prism. The IC_50_ values for bufalin were calculated based on the Hill-Slope model.

### Western Blotting Assays

Cell lysate was prepared by adding RIPA buffer (containing 10 mM Tris-Cl pH 8.0, 1 mM EDTA, 0.5 mM EGTA, 1% Triton X–100, 0.1% sodium deoxycholate, 0.1% SDS, 140 mM NaCl, and protease inhibitors) to cells kept on ice. After spinning samples at 16,000 rpm for 15 minutes at 4°C, insoluble materials in the collected cell lysate were precipitated and discarded. The total protein concentration was measured using a Bradford protein assay. An equivalent mass of each sample was loaded and electrophoretically separated by 10% SDS-PAGE. Then proteins on PAGE the gel were transferred to a PVDF membrane. Anti-SRC–3 (Cell Signaling Technology Co. Cat# 2126) and anti-actin (Cell Signaling Technology Co. Cat# 8457) were used to probe the membranes.

### Single Cell Motility Assays

Microtiter 96-well plates pre-coated with collagen V were covered with beads provided by a Cell Motility HCS kit (Thermo Scientific Co. Cat# K0800011). 500 LM3-3 cells were seeded in each bead-coated well. After 18 hours of incubation, the cell tracks impressed upon the beads were fixed with 4% paraformaledhyde (PFA) and photographed with a phase contrast microscope. The cell track areas (pixels) were counted using Image J. To attain statistical significance, more than 50 cell tracks in each sample were counted.

### Synthesis of 3-Phospho-Bufalin

*3-(Di-t-butyl-phospho)-bufalin*: Bufalin (100 mg, 0.259 mmol) was dissolved in dichloromethane (DCM, 5 mL) under nitrogen. 1-H-tetrazole (3 mL, 0.45 M in CH_3_CN) and di-*t*-butyl diethylphosphoramidite (0.15 mL, 0.518 mmol) were added subsequently. The reaction was stirred at room temperature for 30 min, then cooled to -78°C in a dry ice-acetone bath. m-Chloroperoxybenzoic acid (mCPBA, 134 mg, 0.777 mmol) in DCM (3 mL) was added dropwise. After 30 min, the reaction mixture was diluted with DCM (10 mL), washed sequentially with Na_2_S_2_O_3_ solution, NaHCO_3_ saturated solution, then brine, and dried over anhydrous sodium sulfate. The organic phase was filtered and concentrated under vacuum. The residue was purified by column chromatography on silica gel with hexanes/ethyl acetate eluent (1:2, v/v) to isolate 3-(di-*t*-butyl-phospho)-bufalin as a white solid (105 mg, two steps with a combined 72% yield). NMR analysis of this product:^1^H NMR (400 MHz, CDCl_3_): δ 7.83 (dd, *J* = 9.6, 2.4 Hz, 1H), 7.22 (d, *J* = 2.4 Hz, 1H), 6.25 (d, *J* = 9.6 Hz, 1H), 4.66–4.68 (m, 1 H), 2.44–2.48(m, 1H), 2.15–2.23(m, 1H), 2.00–2.08 (m, 1H), 1.33–1.88 (m, 13H), 1.48 (s, 18H), 1.21–1.40 (m, 7H), 0.94 (s, 3H), 0.70 (s, 3H).

*3-Phospho-bufalin*: 3-(Di-*t*-butyl-phospho)-bufalin (20 mg, 0.035 mmol) was dissolved in a mixture of DCM (14 mL) and methanol (2.8 mL), then added to 4N HCl in dioxane (1.0 mL) at 0°C. After stirring at 0°C for 30 min, the solvent was removed under vacuum to afford the target product 3-phospho-bufalin as a white solid (14 mg, 85% yield). Once the purity of the product was verified (determined >99% using high performance liquid chromatography (HPLC)), it was used without further purification. NMR analysis of this product:^1^H NMR (400 MHz, CD_3_OD): δ 7.99 (d, *J* = 9.6 Hz, 1H), 7.43 (s, 1H), 6.28 (d, *J* = 9.6 Hz, 1H), 4.65 (br, 1H), 2.53–2.56 (m, 1H), 2.11–2.21 (m, 2H), 1.93–2.03 (m, 2H),1.43–1.84 (m, 12 H), 1.21–1.29 (m, 6H), 0.98 (s, 3H), 0.72 (s, 3H); ESI-MS (m/z): [M + H]^+^ calculated for C_24_H_36_O_7_P, 467.2; found, 467.0. ESI-HRMS (m/z): [M − H]^−^ calculated for C_24_H_34_O_7_P, 465.2042; Found, 465.2028.

### Animal Studies

All the animal studies were conducted with the approval of the Institutional Animal Care and Use Committee (IACUC) at Baylor College of Medicine (BCM). All the mice were sacrificed in a CO_2_ chamber. In addition, any animals that showed loss of mobility, weight loss or other symptoms related to tumor growth were euthanized. Tumors were allowed to reach >20% of body weight or ulcerate. All animals were sacrificed and tumors were harvested at no more than 1500 mm^3^ in volume. In addition, any animal that displayed immobility, huddled posture, inability to eat, ruffled fur, self-mutilation, vocalization, wound dehiscence, hypothermia, or greater than 20% weight loss were euthanized as per BCM guidelines.

### Electrocardiogram (EKG) Measurements

Female ICR mice (5 weeks old) were treated with bufalin or 3-phospho-bufalin via intravenous (i.v.) and intraperitoneal (i.p.) injections, respectively, at a range of concentrations. Non-invasive EKG recordings were made using the ECGenie system (Mouse Specifics) at several subsequent time points. All data was collected at a similar hour in the day, as the heart rate is most stable during “inactive” daylight hours [30]. Data acquisition was performed using the program LabChart 6 (AD Instruments). Individual EKG signals were then evaluated using e-MOUSE physiologic waveform analysis software (Mouse Specifics) as previously described [31].

### Pharmacokinetic Studies of 3-Phospho-Bufalin

3-Phospho-bufalin (0.5 mg/kg, i.p.) were injected into ICR mice (n = 3). Twenty microliters of blood was collected via the tail vein at 5, 15, 30 min, and 1, 2, 4, 6, 12, and 24 h.

To calibrate the quantification of bufalin and 3-phospho-bufalin, solutions of each analyte were prepared by serial dilution in 50% acetonitrile in water. Five quality control (QC) samples at 10 ng/mL, 20 ng/mL, 500 ng/mL, 4000 ng/mL and 8000 ng/mL of plasma were prepared independently of those used for the calibration curves. QC samples were prepared on the day of analysis in the same way as the calibration standards.

Standards, QC samples, and unknown samples were injected into LC-MS/MS for quantitative analysis. The pharmacokinetic parameters of p-Buf were analyzed using WinNonLin. The PK trace was fitted into a non-compartmental extravascular model.

### Therapeutic Efficacy of 3-Phospho-Bufalin in an Orthotopic TNBC Mouse Model

To establish orthotopic breast tumors, 0.75 × 10^6^ MDA-MB-231-LM3-3 cells were injected into one of the second mammary fat pads of nude mice (Charles River Laboratories, female, 6–7 weeks, n = 6/group). Phospho-bufalin was dissolved in PBS for tumor treatment. PBS was injected as the vehicle control. When tumors became palpable, generally 14 days after injection, mice were randomized into two groups. The treatment group was then injected with phospho-bufalin subcutaneously (0.75 mg/kg per dose, 3 doses per week) for 3 weeks while the control group received PBS. Tumor lengths and widths were measured three times per week. Tumor sizes were calculated by: (length × width × width)/2.

### Immunohistochemistry

The formalin-fixed, paraffin-embedded tissue was dried at 58°C for 1 hour in an oven, and de-paraffinized by soaking slides in xylene, ethanol, and finally distilled water. Antigen was retrieved by steaming slides in a target retrieval solution, 10 mM citrate buffer (pH 6.0), for 30 minutes. Endogenous peroxidases were blocked with a 3% of hydrogen peroxide solution in PBS buffer. The leftover hydrogen peroxide was removed with PBS rinse. The tissue was blocked with 2.5% of normal serum to reduce nonspecific staining. To stain the SRC–3 antigen, anti-SRC–3 antibody (Mouse source, BD Biosciences Inc. Cat# 611104) was pre-diluted 1:400 in a normal serum blocking solution. To stain the Ki–67 antigen, anti-Ki–67 (Rabbit source, Cell Signaling Technology Inc. Cat#9027) was used in 1:10000 dilution. During the staining procedure, the sections were incubated with HRP-polymer conjugated secondary antibodies (Vector Laboratories, Cat#7401 for rabbit antibody, Cat#MP–2400 for mouse antibody). To visualize the immunostaining, the sections were incubated with a fresh DAB solution (Dako Inc. Cat# K3468) and monitored under a microscope. Once ideal signals were observed, the reaction was stopped by rinsing the slides with water. Nuclear counterstain was also performed by immersing slides in Mayer’s Hematoxylin and a bluing reagent.

## Results

### Overexpression of SRC–3 Correlates with Poor Prognosis in TNBC Patients

**Fig 1:**
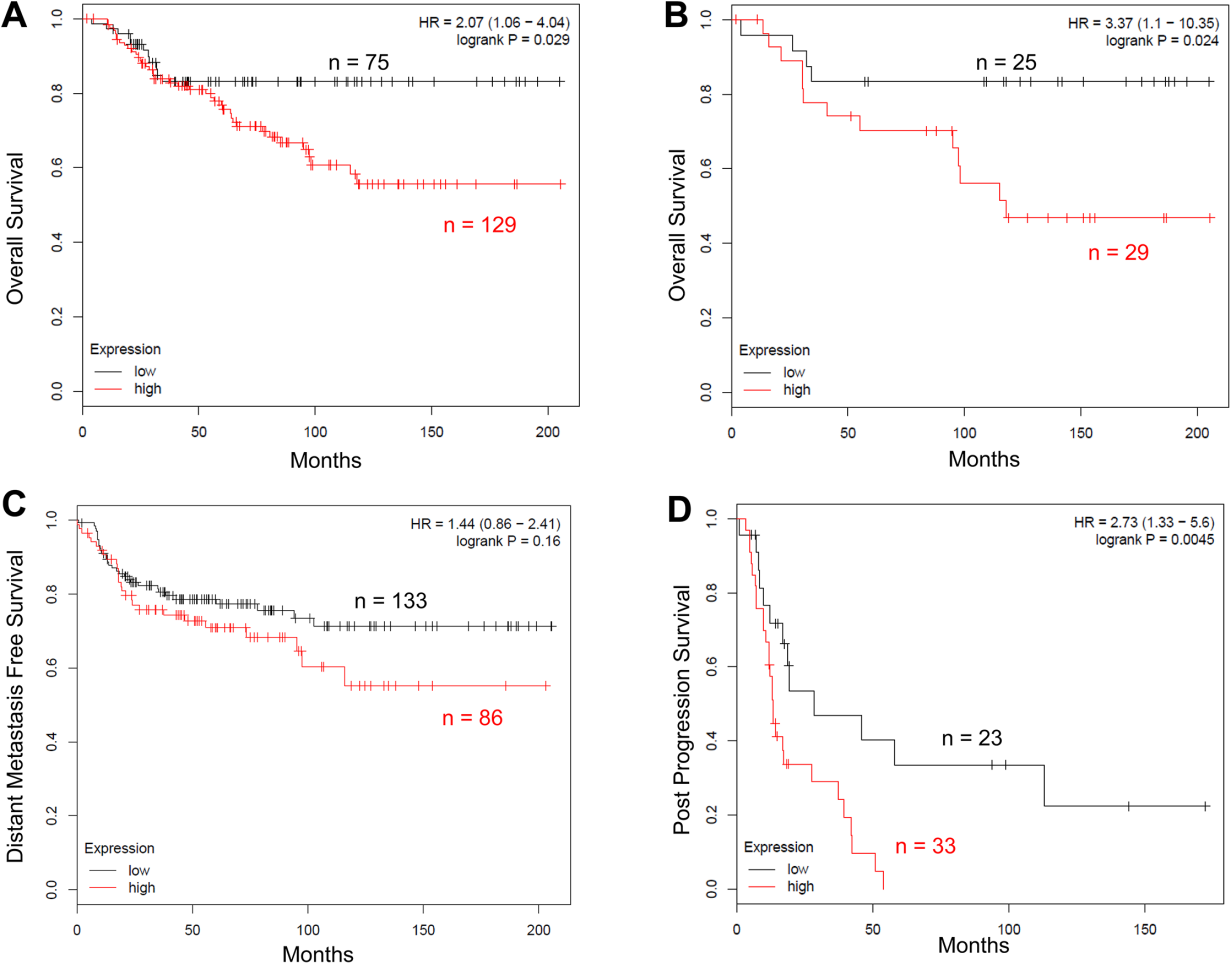
Kaplan-Meier analysis on SRC–3 level and survival rate of TNBC patients. (A) Overall survival in low SRC–3 expression group and high SRC–3 expression group. (B) Overall survival in systemically untreated TNBC patients with low or high SRC–3 expression. (C) Distant metastasis-free survival in low or high SRC–3 expression group. (D) Post-progression survival in low or high SRC–3 expression group. KM-plotter database developed by Gyorffy et al. was used for analyses (51). Gene symbol: NCOA3. SRC–3 JetSet probe was used (Affymetrix ID: 209061_at). Patients were split by auto select best cutoff. Patients with basal-like intrinsic subtype was selected. For statistical significance, Log Rank test was used.

Although SRC–3 levels correlate with clinical endocrine resistance in ER+ breast cancer [20–22], the role of SRC–3 in TNBC has not been investigated in great detail. We retrospectively analyzed KM-plotter microarray data from breast cancer patients comprising basal-like intrinsic subtypes [23], 80–100% of which are generally triple negative [32]. We found that high expression of SRC–3 is inversely correlated with overall survival (OS, Log Rank P = 0.029, HR = 2.07, Fig 1A). A similar trend was observed for the overall survival of systemically untreated TNBC patients (Log Rank P = 0.024, HR = 3.37, Fig 1B). Overexpression of SRC–3 does not correlate with poor distant-metastasis-free survival to a statistically significant level (DMFS, Log Rank P = 0.13, HR = 1.48, Fig 1C). In addition, post-progression survival is markedly lower in patients with high levels of SRC–3 (PPS, Log Rank P = 0.0045, HR = 2.73, Fig 1D). Overall, overexpression of SRC–3 correlates with poor prognosis in TNBC patients.

### Inhibition of SRC–3 by Bufalin Potently Reduces TNBC Cell Viability, but Spares Primary Hepatocytes

**Fig 2:**
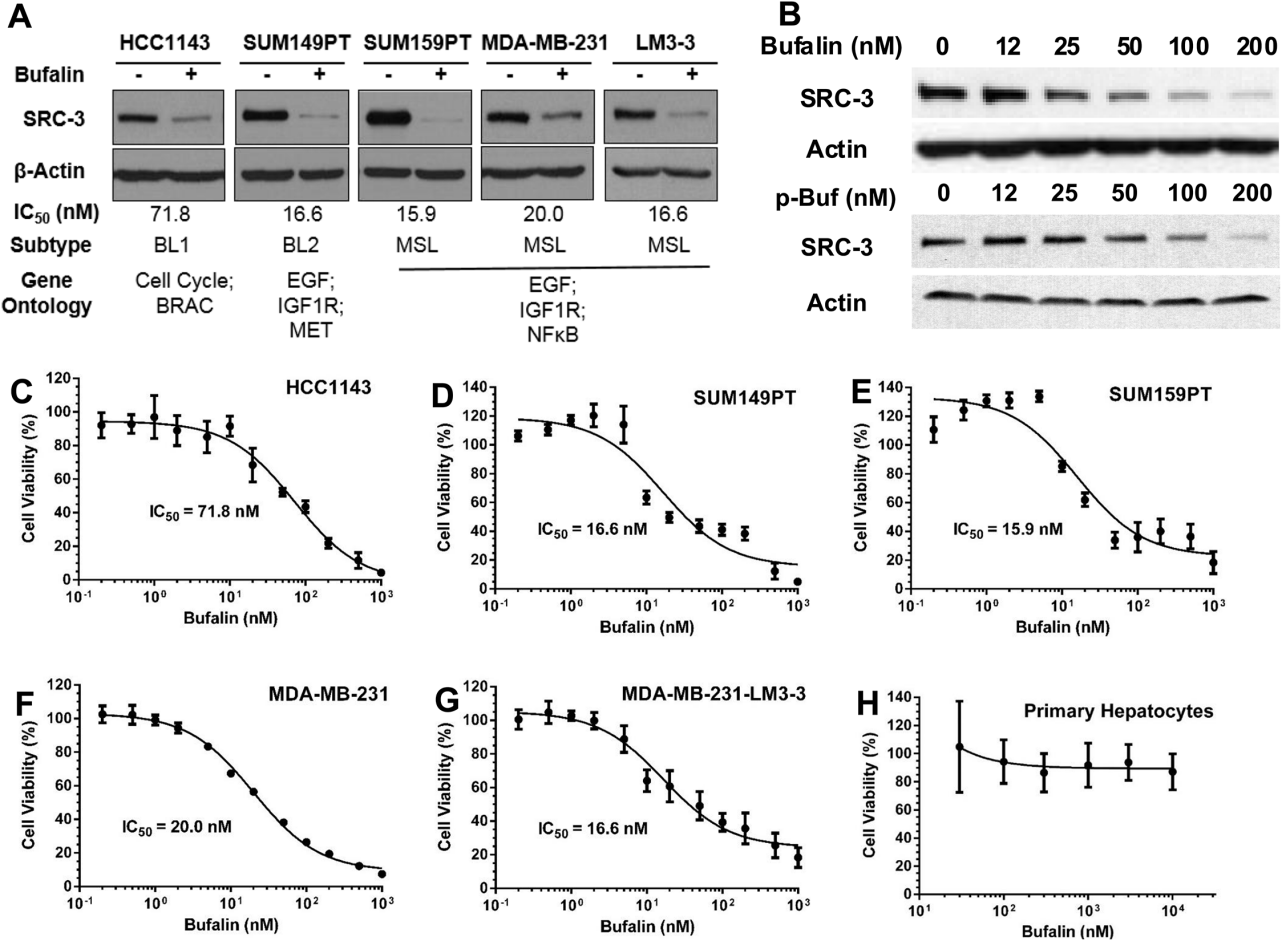
Bufalin downregulates SRC–3 and decreases cell viability in TNBC cells. (A) Western blot shows that bufalin (100 nM) downregulates SRC–3 protein levels in TNBC cells. IC_50_ values, TNBC subtypes and gene ontologies are also listed. (B) Western blotting shows a dose-dependent downregulation of SRC–3 by bufalin and phospho-bufalin in LM3-3 cells. (C-H) Bufalin decrease cell viability of TNBC cells including HCC1143 (C), SUM149PT (D), SUM159PT (E), MDA-MB–231 (F) and MDA-MB-231-LM3-3 (G), but not in primary mouse hepatocytes (H). Data are presented as mean±SD.

We have previously shown that bufalin is a potent small molecule inhibitor of SRC–3 [25]. In the present study, Western blotting results show that 100 nM of bufalin can reduce the SRC–3 protein levels in a panel of TNBC cell lines—HCC1143, SUM149PT, SUM159PT, MDA-MB–231, and MDA-MB-231-LM3-3 (Fig 2A). In addition, bufalin downregulated SRC–3 in MDA-MB-231-LM3-3 in a dose-dependent manner (Fig 2B).

Our previous study showed that low nM concentrations of bufalin can significantly reduce the viability of ER+ breast cancer cells [25]. In this study, TNBC cell lines were treated with a range of bufalin concentrations for 72 h. Cell viabilities were measured and compared against untreated control cells by MTT assay. We found that the growth of all TNBC cell lines was potently inhibited by bufalin with IC_50_ values lower than 20 nM (Fig 2D–2G), with the single exception in HCC1143 where an IC_50_ = 71.8 nM was observed (Fig 2C).

Bufalin is mainly excreted through hepatocyte clearance via a biliary route [33]. Therefore, bufalin’s liver toxicity should be considered during the drug development process. Remarkably, even at concentrations of up to 10 μM, the highest concentration treatment, bufalin did not cause any observable toxicity in mouse primary hepatocytes (Fig 2H), consistent with previous studies [26]. This selective cytotoxicity bodes well for bufalin’s candidacy as a potential anticancer drug.

### Inhibition of SRC–3 by Bufalin Decreases TNBC Cell Motility

**Fig 3:**
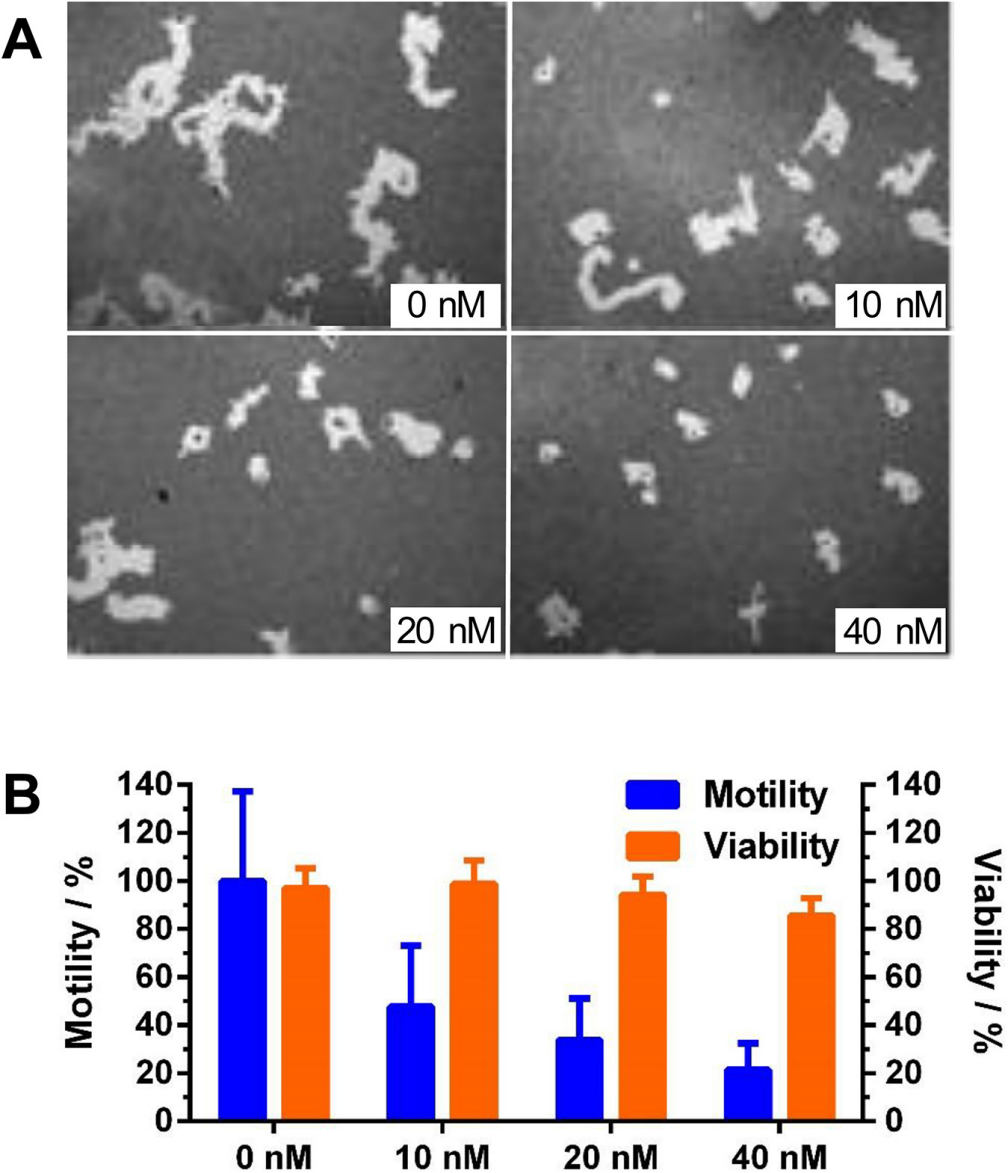
Bufalin downregulates SRC–3 and reduces cell motility in MDA-MB-231-LM3-3. (A) Cell motility assay of LM3-3 cells was performed using a Cellomics cell motility kit. The bright areas of 50 images for each sample were analyzed using Image J. (B) Treatment of LM3-3 cells with different concentrations of bufalin for 12 h showed minimal toxicity but significant motility reduction. Data are presented as mean±SD.

Down-regulation of SRC–3 can inhibit cell motility, invasion, and tumor metastasis [18]. The single cell motility assay showed that the motility of LM3-3 cells decreased with increasing concentrations of bufalin (Fig 3A and 3B). Notably, the experimental conditions used in this assay produced minimal toxicity toward LM3-3 cells at the 12 h time point (Fig 3B), indicating that the observed motility difference was not due to decreased viability.

### Bufalin Synergizes with Gefitinib in Triple Negative Breast Cancer Cells

**Fig 4:**
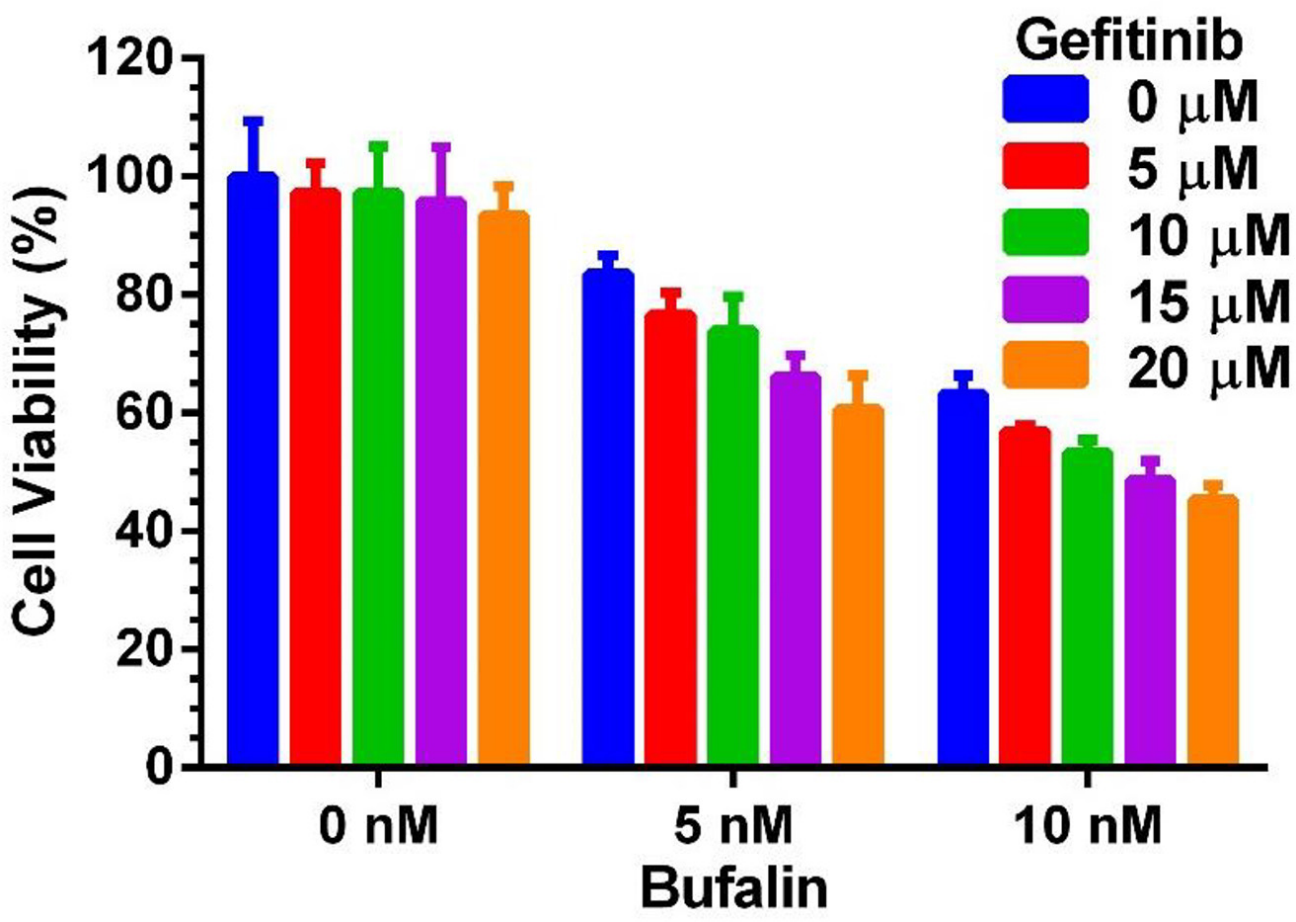
Synergistic effect of Gefitinib and bufalin in TNBC cells. When combined with 5 or 10 nM of bufalin, co-treatment with the EGFR inhibitor gefitinib synergistically reduced LM3-3 cell viability. Data are presented as mean±SD.

Despite the popularity of tyrosine kinase inhibitors (TKIs) in conventional cancer therapies [34–44], none have been found to be successful for the treatment of TNBC [45]. However, over-expression of EGFR is a hallmark of TNBC and usually correlates with poor prognosis [46]. The inability of EGFR inhibitors, such as gefitinib, to arrest TNBC growth has been attributed to crosstalk between growth factors [47]. Despite the fact that gefitinib, at concentrations up to 20 μM caused minimal toxicity in LM3-3 cells, bufalin can synergize with gefitinib in TNBC cells, likely through crosstalk inhibition, given SRC–3’s role as a growth factor signaling integrator (Fig 4).

### Synthesis of 3-Phospho-Bufalin

**Fig 5:**
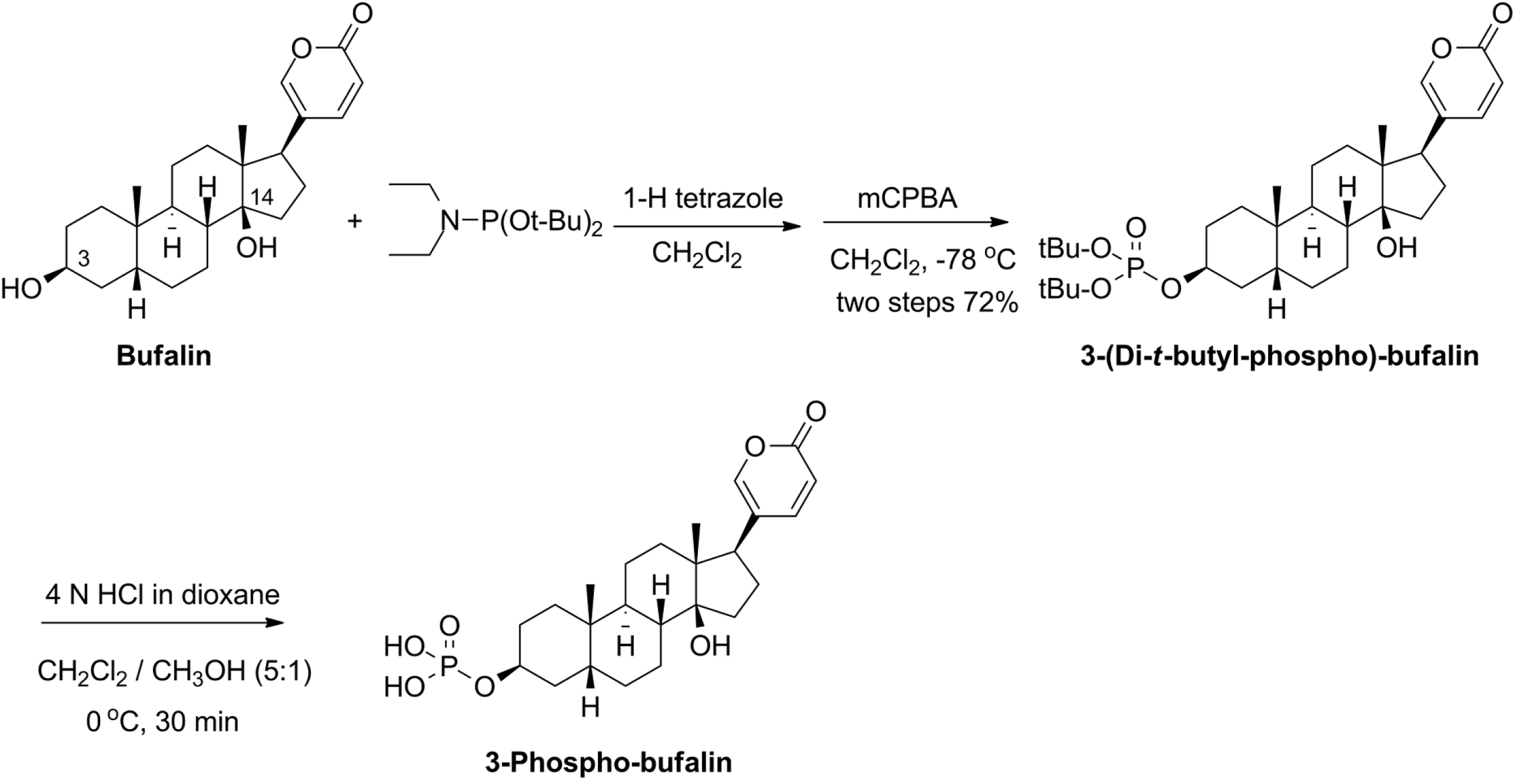
Synthetic scheme of 3-phospho-bufalin.

Bufalin has low solubility in aqueous solutions, which limits its use as a therapeutic agent. Introducing a phosphate group is a common strategy to increase a compound’s aqueous solubility, and has been used before in prodrug development [48]. As shown in Fig 5, bufalin was converted to 3-(di-*t*-butyl-phospho)-bufalin through reaction with di-*t*-butyl diethylphosphoramidite and subsequent oxidation with *m*-chloroperoxybenzoic acid (mCPBA). The *t*-butyl groups were carefully de-protected in an acidic environment to supply the final p-Buf. The phosphate group now can be cleaved by endogenous phosphatases to regenerate free bufalin (see below). Similar to free bufalin, p-Buf can also inhibit SRC–3 expression levels in LM3-3 cells (Fig 2B). It should be noted that p-Buf is a prodrug and requires phosphatases to re-generate bufalin. Phospho-bufalin appeared to be less potent than bufalin to inhibit SRC–3 levels (Fig 2B), which may be due to incomplete conversion of p-Buf to bufalin under the culture conditions.

### 3-Phospho-bufalin Reduces Cardiotoxicity *In Vivo*

Because cardiac glycosides can cause cardiotoxicity, their clinical application necessitates close monitoring. Despite the fact that there are many *in vitro* assays available to predict cardiotoxicity of a drug candidate, *in vivo* electrocardiogram measurements remain the gold standard for this assessment.

Intravenous injection of free bufalin (0.5 mg/kg) caused significant decreases in heart rate and an increase in EKG parameters five min post-injection (Table 2). The changes in heart rate and electrophysiology are similar to the observed cardiotoxicity caused by digoxin [49]. An observed QTc interval prolongation may indicate altered K^+^ or Ca^2+^ ion channel function during repolarization [49]. The cardiotoxicity caused by bufalin (0.5 mg/kg) appears to be transient and most of the electrophysiology data reflected a return to normal cardiac activity 24 h post-injection. Additionally, we decreased the injection dose of free bufalin from 0.5 to 0.1 mg/kg and found no acute cardiotoxicity.

**Table 2:** Electrocardiogram (EKG) measurements in mice treated with bufalin and 3-phospho-bufalin.

	Dose (mg/kg)	Post-injection	HR (BPM)	RR (ms)	QRS (ms)	QT (ms)	QTc (ms)	ST (ms)
**Untreated**	0	- 5 min	799.0 ± 3.0	75.1 ± 0.3	10.4 ± 0.2	37.8 ± 2.1	43.6 ± 2.4	27.9 ± 2.0
		+10 min	805.5 ± 17.7	77.1 ± 5.3	10.1 ± 0.5	39.0 ± 2.2	45.0 ± 1.6	29.5 ± 2.6
**Bufalin**	0.1	- 5 min	746 ± 15.8	82.2 ± 6.9	10.2 ± 0.4	40.2 ± 1.8	44.7 ± 1.1	30.6 ± 1.9
		+ 5 min	724 ± 5.7	88.5 ± 0.4	10.0 ± 0.1	41.0 ± 0.7	44.2 ± 0.6	31.6 ± 0.8
	0.2	- 5 min	769.3 ± 17.2	78.0 ± 1.8	9.2 ± 0.1	41.2 ± 1.8	46.7 ± 1.8	32.5 ± 1.7
		+ 5 min	*500*.*3 ± 24*.*5*	*123*.*6 ± 9*.*5*	10.9 ± 0.6	*55*.*6 ± 10*.*2*	49.8 ± 4.8	*45*.*2 ± 9*.*6*
	0.3	- 5 min	683.3 ± 18.7	89.4 ± 0.6	10.4 ± 1.6	46.1 ± 0.5	48.3 ± 1.3	36.3 ± 1.9
		+ 5 min	*464*.*7 ± 5*.*7*	*131*.*0 ± 0*.*9*	14.4 ± 0.2	*61*.*4 ± 1*.*6*	53.7 ± 1.3	*47*.*5 ± 1*.*3*
	0.5	- 5 min	761.1 ± 10.9	78.9 ± 1.1	11.1 ± 0.2	39.9 ± 1.5	44.9 ± 1.8	29.3 ± 1.5
		+ 5 min	*582*.*2 ± 30*.*5*	*103*.*7 ± 5*.*3*	13.4 ± 0.3	*51*.*4 ± 3*.*0*	50.4 ± 1.6	*38*.*5 ± 3*.*3*
		+ 24 h	766.1 ± 2.5	78.4 ± 0.3	10.7 ± 0.2	41.9 ± 1.1	47.3 ± 1.2	28.7 ± 1.3
**3-Phospho-Bufalin**	7.5	- 5 min	754.7 ± 6.6	79.6 ± 0.7	10.6 ± 0.1	40.2 ± 0.6	45.0 ± 0.5	30.1 ± 0.7
		+ 10 min	788.7 ± 1.2	76.1 ± 0.1	11.0 ± 0.1	38.2 ± 0.9	43.8 ± 1.0	27.6 ± 0.9
	11.25	- 5 min	798.0 ± 12.8	75.8 ± 0.2	10.6 ± 0.3	39.1 ± 2.4	45.1 ± 2.9	29.0 ± 2.2
		+ 5 min	779.7 ± 1.5	77.0 ± 0.1	10.9 ± 0.1	37.1 ± 0.3	42.3 ± 0.4	26.7 ± 0.4
		+ 10 min	761.3 ± 7.5	78.8 ± 0.8	11.1 ± 0.2	38.1 ± 0.6	43.0 ± 0.6	26.6 ± 0.5
		+ 15 min	755.0 ± 1.0	79.5 ± 0.1	11.5 ± 0.1	39.1 ± 0.9	43.9 ± 0.9	28.1 ± 0.8

Electrocardiogram (EKG) measurements in ICR mice treated with different doses of bufalin and 3-phospho-bufalin. EKG was measured 5 min before drug administration and 5, 10, 15 min and 24 h after drug administration. Significant changes in EKG output were highlighted in italics. While bufalin at high does induces acute cardiotoxicity, 3-phospho-bufalin is well tolerated up to 11.25 mg/kg. The measurements were performed in 3 mice. EKG parameters are presented as mean±SD.

**Fig 6:**
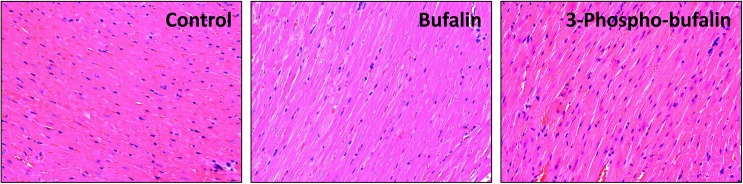
H-E staining of bufalin or 3-phospho-bufalin treated mouse heart. H&E staining of heart tissues 24 h after drug administration. Neither of bufalin or 3-phospho-bufalin caused severe damage to the heart muscle, indicating that the acute toxicity of bufalin is reversible.

In contrast, following 3-phospho-bufalin administration even at 7.5mg/kg and 11.25 mg/kg we did not observe significant changes in EKG parameters. We therefore assume that 3-phospho-bufalin has no acute cardiotoxicity at these higher doses, suggesting it may be a safer agent than its parent drug, bufalin. In addition, histological examinations found little damage to cardiomyocytes treated with bufalin and P-Buf (Fig 6).

### Pharmacokinetics of 3-Phospho-Bufalin

**Fig 7:**
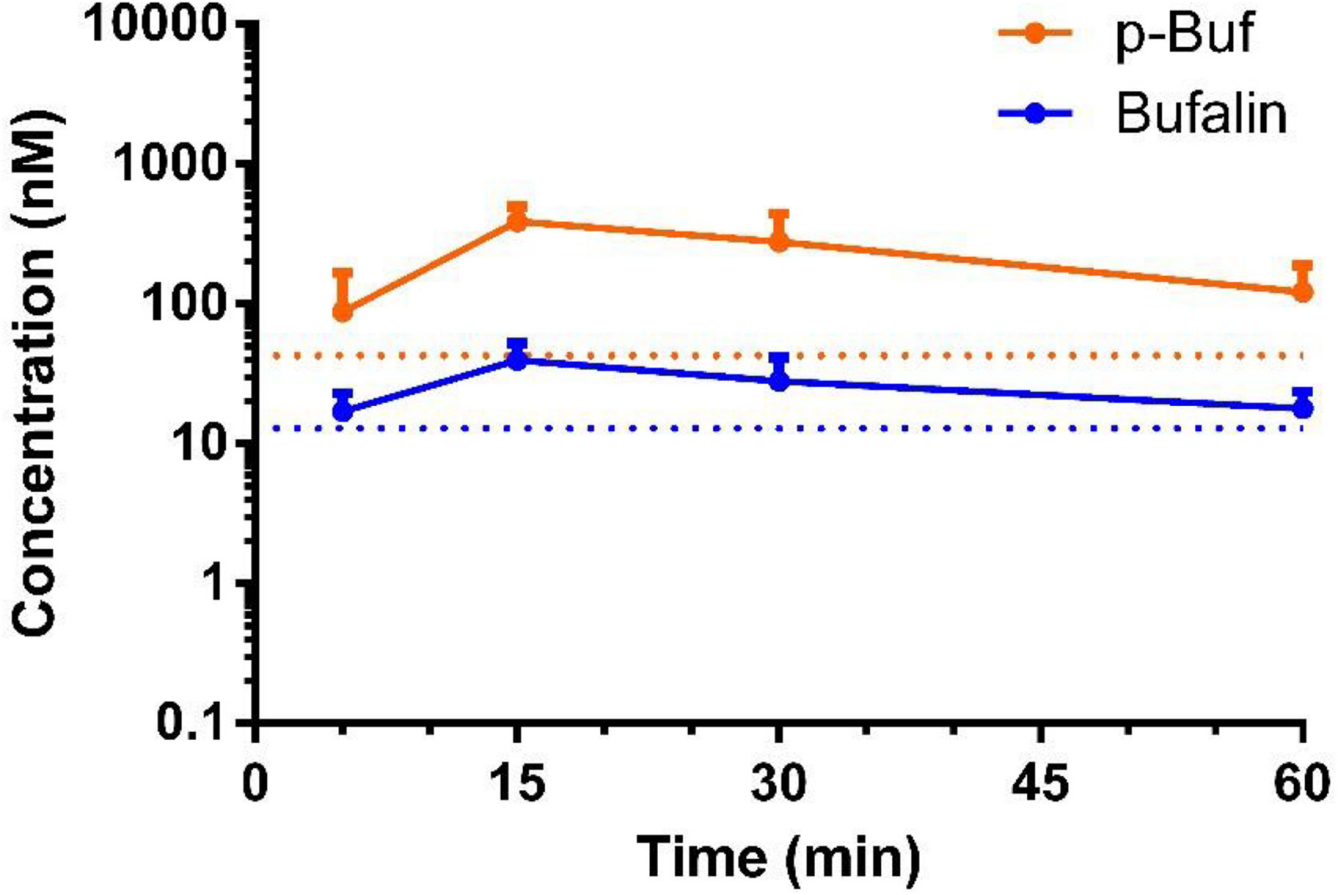
Pharmacokinetics (PK) of 3-Phospho-bufalin (p-Buf). PK trace of p-buf (orange) and free bufalin generated from p-buf (blue) in mice treated with p-buf (0.5 mg/kg). The blue and orange dotted lines represent the lower limit of quantification (LLOQ) for bufalin and p-buf in this assay, respectively.

The pharmacokinetic profile of p-Buf was measured in ICR mice. P-Buf was dissolved in PBS and administered intraperitoneally at a dose of 0.5 mg/kg. Blood (20 μL for each time point) was collected at 5, 15, 30 min, and 1, 2, 4, 6, 12, and 24 h. The plasma concentrations of both p-buf and bufalin that is generated by hydrolysis of p-buf were analyzed using LC-MS/MS. The pharmacokinetic traces for p-buf and released bufalin are shown in Fig 7.

Both p-Buf and free bufalin were detected in plasma during the first hour. Detection of free bufalin in plasma also confirms that p-buf can be converted to parent bufalin *in vivo*. All the pharmacokinetic parameters are summarized in Table 3.

**Table 3:** Pharmacokinetic (PK) parameters of 3-Phospho-bufalin.

**Dose (mg/kg)**	0.5
**t**_**1/2**_ **(h)**	0.742 ± 0.104
**t**_**max**_ **(h)**	0.25
**AUC**_**last**_ (μM*h)	0.031 ± 0.018
**AUC**_**inf**_ (μM*h)	0.046 ± 0.018
**AUC**_**last**_ /D (μM*h*kg/mg)	0.075 ± 0.044

3-Phospho-bufalin (0.5 mg/kg by i.p.) were injected to female ICR mice (n = 3). Blood was collected at 5, 15, 30 min, and 1, 2, 4, 6, 12, and 24 h. The plasma concentrations of bufalin and p-buf were analyzed using LC-MS/MS.

### 3-Phospho-Bufalin Inhibits Tumor Growth in an Orthotopic TNBC Model

**Fig 8:**
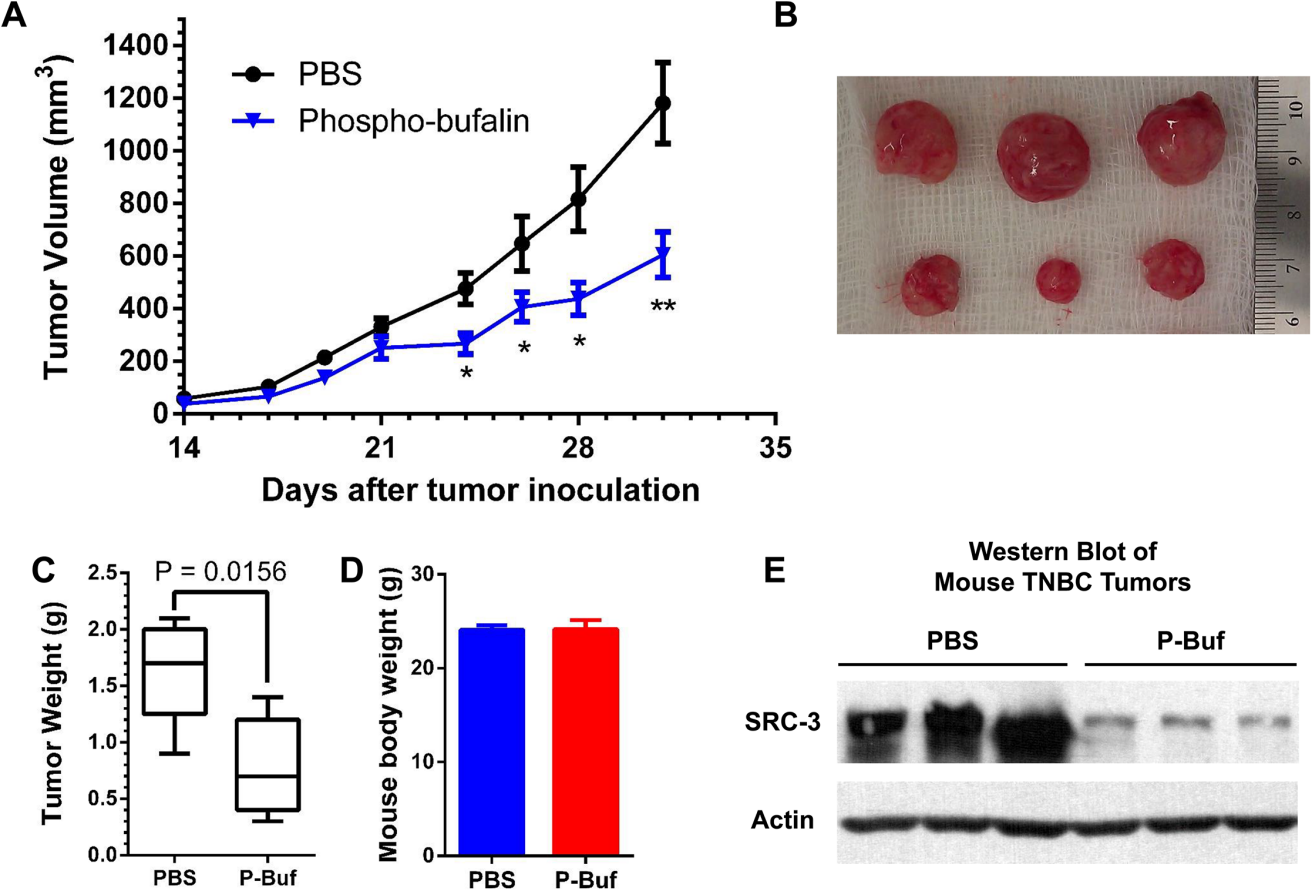
Therapeutic efficacy of 3-phospho-bufalin in an orthotopic TNBC model. (A) LM3-3 cells were inoculated into the mammary fat pads of nude mice (female, 4–5 weeks). The treatment was started 14 days after tumor inoculation. The treatment group was treated with 3-phospho-bufalin (0.75 mg/kg) 3 times per week for 3 weeks (n = 6). The control group was treated with PBS (n = 6). Tumor volumes was measured three times per week. As shown, 3-phospho-bufalin can significant inhibit TNBC tumor growth. Data are presented as mean±SEM. *, P < 0.05; **, P< 0.01, by t-test. (B) Representative images of harvested tumors. (C) Comparison of the tumor weights from both the 3-phospho-bufalin treated group and the PBS treated control group. Data are presented in a box plot with mean, minimum and maximum values. P = 0.0156 by t-test. (D) Body weight of mice at the end of the experiment.(E) Western blotting analyses of the SRC–3 protein levels in the tumor tissues of the p-Buf and PBS treated mice.

**Fig 9:**
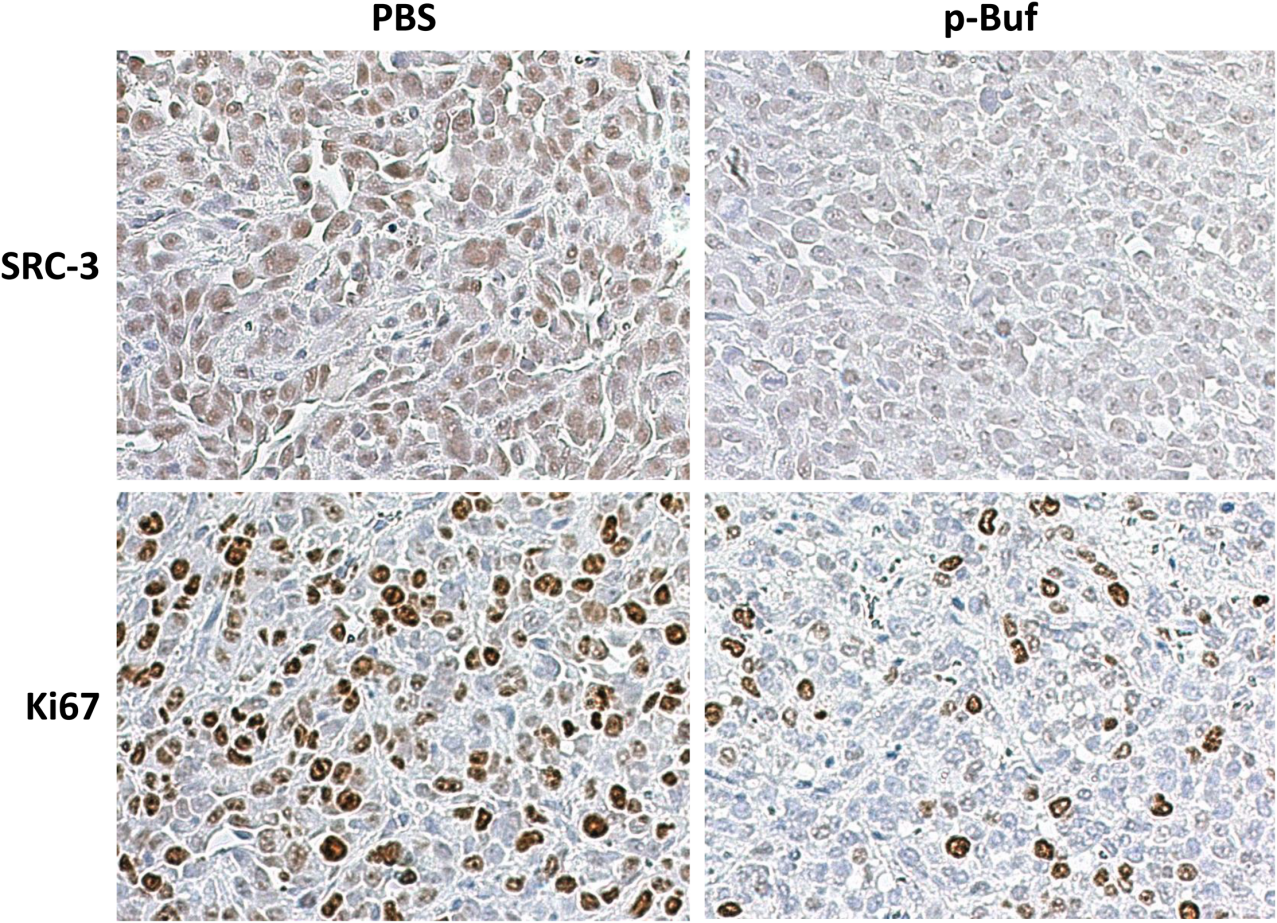
P-buf downregulates SRC–3 levels and reduces the number of Ki–67 positive cells in an orthotopic TNBC model. Multiple tumor tissues (N>3) collected from both control group (administrated with PBS), and p-buf treated group were processed and immunohistochemically stained with anti-SRC–3 antibody (upper panel), or anti-Ki–67 antibody (bottom panel). The staining results were photographed under a microscope. One representative photo for each staining is presented.

The therapeutic efficacy of p-Buf was tested in an orthotopic TNBC model. LM3-3 cells (0.75×10^6^ cells per mouse) were inoculated into the second pair of mammary gland fat pads of nude mice (female, 4–5 weeks). The mice were randomized into two groups based on luciferase imaging (n = 6 per group). Two weeks after tumor inoculation, the treatment group began receiving p-Buf (0.75 mg/kg) three times per week while the control group received PBS. Tumor volumes were measured three times per week. As shown in Fig 8A, p-Buf can significantly inhibit TNBC tumor growth. At day 31, the mice were euthanized and tumors were harvested. The tumors derived from PBS treated mice weighed ~2.4 times more than those of the experimental treatment group (Fig 8B and 8C). At the end of the experiment, both the treatment and control groups had statistically the same body weight, indicating minimal toxicity of phospho-bufalin.To test whether p-Buf can also cause SRC–3 down-regulation in TNBC mouse model, the SRC–3 levels in tumor tissues were detected both by Western blotting and immunohistochemistry (IHC). As shown in Fig 8E, the SRC–3 protein levels determined using Western blotting were significantly lower in the p-Buf treated tumor tissues than in the PBS treated counterparts. Similar to the Western blotting analysis, the SRC–3 level in p-Buf treated tumor tissue was also significantly lower than the PBS treated control group based on IHC staining (Fig 9). Ki–67, as a cell proliferation mark, was also immunochemically analyzed. Accordingly, the p-Buf treated tumor tissue had dramatically fewer Ki–67 positive cells (Fig 9).

## Discussion

### SRC–3 as a Prognostic Marker for Triple Negative Breast Cancer

SRC–3 was first discovered in breast cancer in 1997 and originally called AIB1 (“amplified in breast cancer 1”) [50]. Its role has been extensively described in ER+ breast cancer. Overexpression has been linked to clinical and experimental endocrine resistance and poor prognosis for ER+ breast cancer [20–22,51]. Amplification and over-expression of SRC–3 also has been observed in many other cancers, including bone [52], gastric [53], prostate [54], ovarian [55], bladder [56] and pancreatic [57] cancers. However, to the best of our knowledge, SRC–3 has not been explored as a prognostic marker for TNBC.

In this study, we found that high expression of SRC–3 is correlated with poor overall survival, poor post-progression survival, and possibly poor distant metastasis-free survival in TNBC patients. Our retrospective analysis is based on the KM-plotter microarray data of breast cancer patients with a basal-like intrinsic subtype. In future studies, we plan to use a tissue microarray (TMA) of TNBC tumors to further investigate the relationship between the SRC–3 protein levels and TNBC patient prognoses.

### Bufalin Inhibits TNBC Cell Proliferation and Migration but Spares Primary Hepatocytes

As a master regulator of cellular growth and development, SRC–3 plays important roles in tumor growth and metastasis. In this study, we found that inhibition of SRC–3 with low nM doses of bufalin can significantly reduce the viability of TNBC cells. It is worthy to note that in all tested TNBC cell lines except HCC1143 (IC_50_ = 71.8 nM), bufalin can reduce cell viability with IC_50_ values below 20 nM. Pietenpol et al. identified six subtypes of TNBC based on their gene expression profiles and ontologies: two basal-like (BL1 and BL2), an immunomodulatory (IM), a mesenchymal (M), a mesenchymal stem-like (MSL), and a luminal androgen receptor (LAR) expressing subtype [58]. Interestingly, both bufalin sensitive subtypes BL2 and MSL display gene ontologies involving growth factor signaling, including the EGF and IGF1R pathways [58]. The BL1 subtype HCC1143, whose top gene ontologies are enriched in cell cycle and cell division pathways, is less sensitive to bufalin induced cell death. This observation is consistent with SRC–3’s known role in many growth factor signaling pathways [59]. In future studies, we will use the ATCC TNBC cell line panel to determine which subtype of TNBC is most susceptible to bufalin treatment.

Down-regulation of SRC–3 via RNA interference (RNAi) leads to reduced tumor metastasis and sensitizes cancer cells to alternative chemotherapies. Naora et al. demonstrated that inhibiting SRC–3 expression in ovarian cancer cells can significantly reduce cell spreading and migration [60]. Minna et al. reported that SRC–3 knockdown renders tyrosine kinase inhibitor resistant lung cancers more sensitive to gefitinib [61]. In addition, SRC–3 knockdown restores sensitivity to tamoxifen in resistant ER+ breast cancer cells [62–64]. In this study, we found that inhibition of SRC–3 can significantly reduce the motility of LM3-3 cells. In addition, bufalin treatment can sensitize TNBC cells to gefitinib, an EGFR inhibitor. We have published previously that SRC–3 cooperates with EGFR to activate focal adhesion kinase (FAK) which promotes cell migration [65]. EGFR mediated signaling caused increased SRC–3 phosphorylation which may explain why co-inhibition of SRC–3 and EGFR can improve treatment outcomes [65].

Because bufalin is mainly metabolized through hepatocyte clearance and through the biliary route, its liver toxicity is a potential issue (33). Remarkably, even at concentrations of up to 10 μM, the treatment maximum, bufalin did not cause any observable toxicity in mouse primary hepatocytes confirming previous observations that it can be innocuous under such conditions [26]. All these *in vitro* data validates bufalin’s potential for anticancer drug development.

### 3-Phospho-Bufalin Prodrug Reduces Cardiotoxicity, Releases Bufalin *In Vivo*, and Inhibits Orthotopic TNBC Tumor Growth

Bufalin has poor aqueous solubility, hindering its clinical use. P-Buf developed in this study is a prodrug derivative whose charged phosphate group significantly enhances its aqueous solubility.

Depending on the dosage, intravenous injection of free bufalin may cause acute cardiotoxicity. In our study, a dose higher than 0.2 mg/kg decreased heart rate and changed EKG parameters. These physiological responses are similar to those of digoxin induced cardiotoxicity. In contrast, 0.1 mg/kg of bufalin did not alter EKG parameters (Fig 6). Therefore, the maximum non-toxic dose must be between 0.1 and 0.2 mg/kg.

Intraperitoneal injection of P-Buf, even at elevated doses (11.25 mg/kg), did not demonstrate appreciable cardiotoxicity as determined by EKG measurements. In the pharmacokinetic study of p-Buf, plasma bufalin concentration crested at 15 min post injection. After a 0.5 mg/kg dose of p-Buf, the plasma C_max_ of bufalin was 0.040 μM. It should be cautioned that rodents are more tolerant of cardiac glycosides than humans, so in the case of any advancement towards clinical applications, we will need to test the maximum tolerated dose of bufalin in rabbits and dogs, which exhibit greater sensitivity to cardiac glycosides [26].

In an orthotopic triple negative breast cancer model, p-Buf can significantly inhibit tumor growth. The therapeutic efficacy of p-Buf is equivalent to that of bufalin nanoparticles in the same model [24]. Based on our PK studies, bufalin was expediently metabolized, becoming undetectable 1 h post injection. Because of its shortened half-life limitations for antitumor therapy, our future research will aim to identify and alter the metabolic pathways of bufalin and/or to modify its structure so as to prolong *in vivo* exposure.

In summary, we demonstrated that SRC–3 can be used as a prognostic marker of TNBC. Inhibition of SRC–3 with bufalin can significantly reduce the viability and motility of TNBC cells. We also developed p-Buf as a prodrug of bufalin to enhance *in vivo* administration and demonstrated its effectiveness in inhibiting tumor growth in an orthotopic TNBC mouse model. Our results suggest the potential for advanced TNBC treatment using targeted therapy with p-Buf.
